# β-Tubulin Isotype, TUBB4B, Regulates The Maintenance of Cancer Stem Cells

**DOI:** 10.3389/fonc.2021.788024

**Published:** 2021-12-23

**Authors:** Dhrishya Dharmapal, Athira Jyothy, Amrutha Mohan, P. G. Balagopal, Nebu Abraham George, Paul Sebastian, Tessy Thomas Maliekal, Suparna Sengupta

**Affiliations:** ^1^ Cancer Research, Rajiv Gandhi Centre for Biotechnology, Thiruvananthapuram, India; ^2^ Department of Biotechnology, University of Kerala, Thiruvananthapuram, India; ^3^ Manipal Academy of Higher Education, Manipal, India; ^4^ Surgical Oncology, Regional Cancer Centre, Thiruvananthapuram, India

**Keywords:** TUBB4B (Tubulin β-2C), cancer stem cells, CSC niche, ephrin-B1, ALDH1A1

## Abstract

Recent advancements in cancer research have shown that cancer stem cell (CSC) niche is a crucial factor modulating tumor progression and treatment outcomes. It sustains CSCs by orchestrated regulation of several cytokines, growth factors, and signaling pathways. Although the features defining adult stem cell niches are well-explored, the CSC niche is poorly characterized. Since membrane trafficking proteins have been shown to be essential for the localization of critical proteins supporting CSCs, we investigated the role of TUBB4B, a probable membrane trafficking protein that was found to be overexpressed in the membranes of stem cell enriched cultures, in sustaining CSCs in oral cancer. Here, we show that the knockdown of TUBB4B downregulates the expression of pluripotency markers, depletes ALDH1A1^+^ population, decreases *in vitro* sphere formation, and diminishes the tumor initiation potential *in vivo*. As TUBB4B is not known to have any role in transcriptional regulation nor cell signaling, we suspected that its membrane trafficking function plays a role in constituting a CSC niche. The pattern of its expression in tissue sections, forming a gradient in and around the CSCs, reinforced the notion. Later, we explored its possible cooperation with a signaling protein, Ephrin-B1, the abrogation of which reduces the self-renewal of oral cancer stem cells. Expression and survival analyses based on the TCGA dataset of head and neck squamous cell carcinoma (HNSCC) samples indicated that the functional cooperation of TUBB4 and EFNB1 results in a poor prognosis. We also show that TUBB4B and Ephrin-B1 cohabit in the CSC niche. Moreover, depletion of TUBB4B downregulates the membrane expression of Ephrin-B1 and reduces the CSC population. Our results imply that the dynamics of TUBB4B is decisive for the surface localization of proteins, like Ephrin-B1, that sustain CSCs by their concerted signaling.

## Introduction

Cancer stem cells (CSCs) are a minority population of cells in a tumor microenvironment, analogous to normal adult stem cells, which can self-renew and differentiate into the different clones in bulk tumor tissue (1, 2). They have been reported to be decisive in the initiation, propagation, multilineage differentiation, relapse, and drug resistance in various cancers (3, 4). The cross-talk between the cells present in the niche of a CSC (CSC niche) that consist of the CSCs, bulk tumor cells at different stages of differentiation, cancer associated fibroblasts, endothelial cells, inflammatory and immune cells help in the maintenance of the tumor. As we have recently proposed, Cancer stemness is a state with high plasticity regulated by the niche factors, including the cytokine levels, hypoxia, acidic pH, cancer-associated fibroblasts, and the signaling pathways initiated thereby (5). Hypoxia and the other biophysical parameters generated in the CSC niche activate several signaling pathways, like Notch, BMP, WNT, JAK/STAT, TGF-β, Hedgehog and Hippo, that in turn up-regulate vital molecules involved in the regulation of stemness, including the pluripotency markers, CD133, ABCG2 and ALDH (5, 6).

Despite the widely accepted concept of CSC niche, the mechanisms by which the concerted regulation of self-renewal and differentiation take place in the CSC niche is not well-characterized. But there are a few reports on the regulation of stem cell niches in normal counterparts, like intestinal crypts. A gradient expression of WNT, originating from the base of the crypt, has been shown to be regulating the stem cell compartment homeostasis (7, 8). Further analysis has shown that a synchronized activation of both WNT and BMP gradients fine-tune the process (9, 10). Another class of proteins that regulate intestinal crypt stem cell fate is the one involved in Eph/Ephrin signaling. WNT-mediated generation of a reverse gradient of Eph receptors and Ephrin-B1 has shown to be critical in maintaining the balance of self-renewal and differentiation in intestinal crypts (11). An immunohistochemical correlative analysis in Glioblastoma has given some insights into the generation of a CSC niche around the CSCs. They have shown that hypoxia-mediated HIF1α expression, along with the expression of GLUT1 and CD44 in the hypoxic pseudopalisading cells, which is one of the several distinct niches for CSCs, enriches CSCs expressing CD133, Sox2, and ALDH1 (12). GLUT1 is a cell surface protein involved in glucose transport, known to regulate the maintenance of CSCs exclusively, without altering their proliferative capacity, possibly suggesting its role in constituting a CSC niche (13). Recently, another study in Glioblastoma has shown that GLUT1 binds to TUBB4, and the trafficking by TUBB4 is critical for its activity to support CSCs (14). Thus, TUBB4B might be an essential molecule regulating the generation of gradients of membrane-bound proteins in CSC niches or normal stem cell niches.

TUBB4B (Tubulin β**-**2C**)** is a β-tubulin isotype, a member of the tubulin family that form microtubule networks, a component of the cell cytoskeleton. Microtubules maintain cell morphology, regulate directional cell motility, cell division, and intracellular transport (15–19). According to The Human Protein Atlas database http://www.proteinatlas.org/, TUBB4B is a prognostic marker in thyroid, endometrial, and liver cancers. The up-regulation of this protein is reported in Prostate cancer, Ovarian cancer, and Glioblastoma (14, 20, 21). Although the expression of TUBB4B is up-regulated in the colorectal cancer metastatic lesions found in sentinel lymph nodes, its downregulation is shown to be essential for the initiation of EMT, a prerequisite for metastasis (22, 23). TUBB4B is also reported to affect cell polarity and regulate focal adhesions in colon cancer cells (23). Though its role in CSC regulation is not well explored so far, its close relative TUBB3 is reported to regulate CSCs in clear cell renal cell carcinoma (24).

The present study aims to investigate the role of TUBB4B in the maintenance of CSCs in oral cancer. Our previous study (data not shown) had shown that TUBB4B is enriched in the membrane fractions of cells maintained in sphere culture, a condition enriching CSCs. Based on that result, we investigated whether TUBB4B regulates CSCs using *in vitro* CSC reporter system, pluripotency markers, *in vitro* serial dilution spheroid formation assay, *in vivo* serial dilution xenograft assay, and immunohistochemical analysis of oral squamous cell carcinoma (OSCC) samples. We also attempted to understand how a tubulin protein, which does not directly regulate any signaling pathway or perform any transcriptional regulation, controls CSC maintenance. Here, we provide evidence of TUBB4B-mediated regulation of Ephrin-B1 localization that supports the CSC niche in oral cancer.

## Materials and Methods

### Reagents

HEPES, PMSF, IPTG, Protease inhibitor cocktail, Poly HEMA, TUBB4B inducible shRNA lentiviral particles, and non-target shRNA lentiviral particles were purchased from Sigma-Aldrich (St. Louis, Missouri, USA). OptiMEM, DMEM, FBS, 100X Insulin selenium transferring, N2 supplement, and 100X penicillin-streptomycin were from GIBCO (Waltham, Massachusetts, USA). Lipofectamine 3000 and Dynabeads Protein A agarose kit was from Invitrogen (Carlsbad, California, USA). OCT was purchased from Thermofischer scientific (Waltham, Massachusetts, USA). ECL reagent was from GE Healthcare Life Sciences (Chicago, IL, USA). TUBB4B siRNA and control siRNA were procured from Santa Cruz (Dallas, Texas, USA). Human EGF and basic FGF were from Cell Signaling Technologies (Danvers, Massachusetts, USA). Puromycin and G418 were obtained from *Invivo*gen. Membrane extraction kit was purchased from Biovision (#K268-50). DAPI- Fluoramount G was purchased from Electron Microscopy Sciences (Hatfield, UK). *In vivo* Luciferin was purchased from Promega (Madison, Wisconsin, USA). Isoflurane I.P. was purchased from a medical pharmacy.

### Antibodies

Primary Antibodies: Anti TUBB4B (WB-1:500; IF, IHC-1:200), Ephrin-B1(WB-1:500; IF, IHC-1:100) and Ephrin-B1-Alexa680(IF, IHC-1:50) were from Santa Cruz Biotechnology (Dallas, Texas, USA); GAPDH (WB-1:1000) antibody was from Thermo Scientific, USA and Mouse IgG isotype control, Rabbit IgG isotype control and Na^+^K^+^-ATPase (1:1000) was from Cell Signaling Technologies (Danvers, Massachusetts, USA); ALDH1A1(IF, IHC-1:50) and Veri-blot IP detection antibody(1:200) were from Abcam (Cambridge, United Kingdom).

Secondary antibodies: Anti-Mouse HRP (A-3673) (1:5000), Anti-Rabbit HRP (A-6154) (1:5000) were procured from Sigma-Aldrich (St. Louis, Missouri, USA). Anti-Mouse Alexa Fluor 488 (1:500), Anti-Mouse Alexa Fluor 568 (1:500) were from Invitrogen.

### Cell Culture

Oral Cancer cell lines HSC-3, HSC-4 and SAS were obtained from the central cell repository, Rajiv Gandhi Centre for Biotechnology. As most of the experiments were performed with HSC-3 cell line, the authenticity of this line was confirmed by STR analysis. A PCR test was done to verify that the cells were free of mycoplasma. The cells were maintained in Dulbecco’s modified eagle’s medium containing 10% fetal bovine serum.

### Sphere Culture Preparation

In a 100 mm dish coated with poly HEMA, 4X10^6^ cells were seeded in sphere media, containing N2 supplement, Insulin selenium transferrin, 20 ng/ml human EGF and 20 ng/ml basic FGF in OptiMEM with phenol red. Spheres were allowed to grow for six days.

### TUBB4B Knockdown

For siRNA transfections, lipofectamine 3000 was used according to the manufacturer’s protocols. 80 nM concentration of TUBB4B siRNA was used for downregulation. To perform lentiviral knockdown, cells were seeded at a confluency of 70-80% in a 96-well plate. Twenty hours post seeding, media was replaced with media containing 8ug/mL polybrene. Viral particles were added to an MOI of 10 and incubated at 37°C 5%CO2 incubator. The media was changed the next day. Forty-eight hours post-infection, the cells were seeded into a T25 flask, and the stable cells were selected with Puromycin (400 ng/mL). The selection was continued till cells in the control un-transduced flask were eliminated entirely. For shRNA induction, cells were treated with 50 mM IPTG for six days.

### Membrane Protein Extraction

Total membrane protein extraction was carried out using BioVision’s membrane protein extraction kit, according to the manufacturer’s protocol. Cells were scraped in PBS and homogenized in the lysis buffer provided using Dounce homogenizer. The supernatant was collected post an initial spin at 700g for 10 minutes. The supernatant was centrifuged at high speed to obtain the membrane fraction as a pellet, which was solubilized in 0.5% Tween in PBS for western blotting.

### *In Vitro* Sphere Formation Assay

Serial dilutions of control and downregulated cells (10,000 to 250 per well) with multiple replicates were plated in 24-well ultra-low attachment plates in sphere media for 6 to 10 days, and sphere formation was monitored. Well-formed spheres were counted under a phase-contrast microscope.

### Immunofluorescence

Cells were seeded in washed coverslips placed in 35mm dishes and grown for 24 hours. Cells were washed with PBS and fixed with 4% Paraformaldehyde for 10 minutes at room temperature. The coverslips were washed with PBS, and cells were blocked without permeabilization in PBS with 5% fetal bovine serum for 1 hour and then incubated with the primary antibodies diluted in blocking buffer overnight at 4°C. Cells were washed thrice with PBS and incubated with secondary antibodies diluted in PBS for 1 hour at room temperature followed by three washes in PBS and mounted on a glass slide with DAPI- Fluoromount G. Confocal Images were acquired using either Olympus FV3000 or Nikon A1R. Immunofluorescence intensities were measured using ImageJ software by drawing an ROI (Region of interest) around the cells.

### Immunohistochemistry

OSCC patient samples were collected from Regional Cancer Centre, Thiruvananthapuram, after getting informed consent from the patients. The study was approved by the Institute Human Ethical Committee of both Rajiv Gandhi Centre for Biotechnology (IHEC/1/2011/04) and Regional Cancer Centre (HEC/24/2011). The OSCC sample details are provided in **Supplementary Table 1**. Tissues fixed in PFA were left in 30% sucrose and embedded in OCT. Cryo-sections of 5µm were heated in citrate buffer at 95°C for 20 minutes for antigen retrieval, blocked, and stained, similar to immunofluorescence protocol. Hematoxylin and eosin staining was performed on the OSCC section immediately post immunofluorescence imaging. The slides were incubated in PBS to dissolve the mountant. The sections were stained with Hematoxylin and Eosin, followed by dehydration and mounted with DPX. Brightfield images were captured.

### Western Blotting

Whole-cell lysates were prepared by incubation in RIPA lysis buffer with protease inhibitor cocktail for 45 minutes on ice, scraped off the plate, spun at 13,200 rpm for 15 minutes, and the supernatants were collected. Thus obtained lysates were run on a 10% SDS PAGE and later transferred onto a PVDF membrane. The membranes were blocked and incubated with primary antibody overnight at 4°C followed by HRP conjugated secondary antibody for 1 hour at room temperature and developed using ECL reagents.

### Immunoprecipitation

Whole-cell lysates were prepared by incubation with Phospho-lysis buffer containing 10% NP-40, 10% glycerol, 137 mM NaCl, 20 mM Tris-HCl (pH 7.4), 20 mM NaF, 1 mM sodium pyrophosphate, 1 mM sodium orthovanadate, 1% Triton X-100, and 5 mM PMSF in the presence of protease inhibitor cocktail for 1 hour in ice, scraped off the plates and centrifuged at 13200 rpm and supernatants were collected. Precleared lysates containing 1-3mg total protein were incubated with primary antibodies (7.5µg) overnight at 4°C. Post incubation, the antibody- lysate mixture was incubated with protein A magnetic beads for 2 hours at 4°C. The beads were washed, eluted, and mixed with 1X loading dye, boiled at 95°C for 5 minutes, and subjected to western blotting.

### Flow Cytometry Analysis

TUBB4B antibody and Ephrin-B1 antibody were conjugated with Alexa flour 488 and 680, respectively. Antibody and Alexa dye were mixed in a ratio of 1:10 in bicarbonate buffer (pH 8.3) in a 96 well plate with stirring for an hour at room temperature. The mixture was run through a Sephadex G-25 column, and small volume fractions were collected by elution with Milli-Q water. Optical densities at 280 and 488 (or 680) were measured to select fractions with maximum conjugation. The fractions were lyophilized and resuspended in 100µl PBS. Conjugated antibody usage dilution was standardized by immunofluorescence microscopy. Monolayer and sphere culture cells were trypsinized, resuspended in 10% DMEM and incubated in 37°C incubator for 1 hour for surface protein re-expression, fixed with 4% PFA for 10 minutes, and stained with the tagged primary antibodies (dilution 1:60 for TUBB4B-Alexa488 and 1:40 for Ephrin-B1-Alexa680) for 1 hour at room temperature and analyzed by FACS ARIA II. The data were analyzed using FACSDiva software v8.0.3. For ALDH1A1-DsRed2 expressing cells, analyses were done straight after trypsinization. Ten thousand cells were counted for each sample.

### Aldefluor Assay

Aldefluor assay was carried out using Aldefluor kit from Stemcell Technologies. The assay was performed according to manufacturers protocol. Four tubes with 2x10^5^ALDH1A1 DsRed2 cells were resuspended in Aldefluor assay buffer and mixed with equal volumes of Aldeflour reagent. DEAB, inhibitor of ALDH activity was added to one of the tubes. The tube contents were mixed well and incubated at 37°C for 40 minutes while mixing every 10 minutes. Excess reagent was washed off after incubation and the cells were analysed by flow cytometry. ALDH1A1-DsRed2 high (ALDH1A1-DsRed2^+^) and ALDH1A1-DsRed2 low (ALDH1A1-DsRed2^-^) cells were gated and checked for Aldefluor activity.

### RNA Seq and Patient Survival Data Analysis

Gene-level transcription estimates of HNSCC patients, as in log2(x+1) transformed RSEM normalized count was obtained from gene expression RNAseq–IlluminaHiSeq dataset of TCGA. The normalized count was plotted for 521 HNSCC patients and 44 normal samples and analyzed for significant difference between means by unpaired t-test. Pearson Correlation analysis was performed between the normalized counts of *EFNB1* and *TUBB4* of the 521 HNSCC patient samples. Pearson r value below 0.5 was considered to have negligible correlation. The normalized count ranged from 3.4 to 10 for TUBB4, whereas that of EFNB1 ranged from 8.3 to 13.9. For survival probability analysis and making Kaplan-Meier plots, patients with a normalized count of *TUBB4* below 6.0 were considered as *TUBB4^lo^*, and values above 7.5 were considered as *TUBB4^hi^.* Patients with a normalized count of *EFNB1* below 11.5 were considered as *EFNB1^lo,^* and the values above 12.5 were considered as *EFNB1^hi^.* The significance of the difference between the groups was computed using Log Rank p-value analysis.

### Xenograft Tumor Generation and Bioimaging

NOD.CB17-*Prkdcscid*/J mice of the age 1.5 to 2 months were used for the experiments. All the animal experiments were approved by the Institute Animal Ethics Committee of Rajiv Gandhi Centre for biotechnology (IAEC number: IAEC/731/SUP/2019). The experiments were conducted according to ARRIVE guidelines. The mice were handled and housed in conventional plastic cages and maintained on an automatic 12 h lighting cycle at a temperature of 22-24°C.

For xenograft generation, HSC-3 cells stably expressing luciferase and IPTG- inducible TUBB4B shRNA were used. The cells were treated with 50mM IPTG for 15 days to induce the expression of the shRNA. The downregulation was confirmed by western blotting prior to injection. Different sets of animals were injected with 10^6^, 10^5^, or 10^4^ cells. IPTG induction was continued by IP injection at a dosage of 9 mg/animal for another seven days.

Animals were imaged using IVIS SPECTRUM *in vivo* imaging system under 3% isoflurane anesthesia. For luminescence acquisition, 150 mg/kg D-Luciferin was injected intraperitoneally 10 minutes before image acquisition. The luminescence was measured with an exposure time of 8 seconds, the output of which comes in photons/sec. For analysis, ROI was drawn on the flanks of the tumor, where the cells were injected. The ROI size was decided by the largest tumor size. The background subtraction was based on images acquired without the administration of the substrate (prescan). The calculations were done using IVIS SPECTRUM imaging software Living Image, Version 4.5.2.18424.

### Analysis of Data

The blots were quantified by densitometric analysis using Bio-Rad’s Quantity One software, and they were normalized with GAPDH for whole-cell lysates or Na^+^K^+^-ATPase for membrane fractions. For fluorescent imaging, events were counted manually, or a region of interest was drawn around cells to measure the fluorescence intensities. For statistical analysis and graph preparations, GraphPad Prism was used. Student’s t-test was used for finding the statistical significance, and p ≤ 0.05 was considered as statistically significant.

## Results

### β-Tubulin Isotype, TUBB4B Is Essential for the Self-Renewal Ability of Oral Cancer Cells

**Figure 1:**
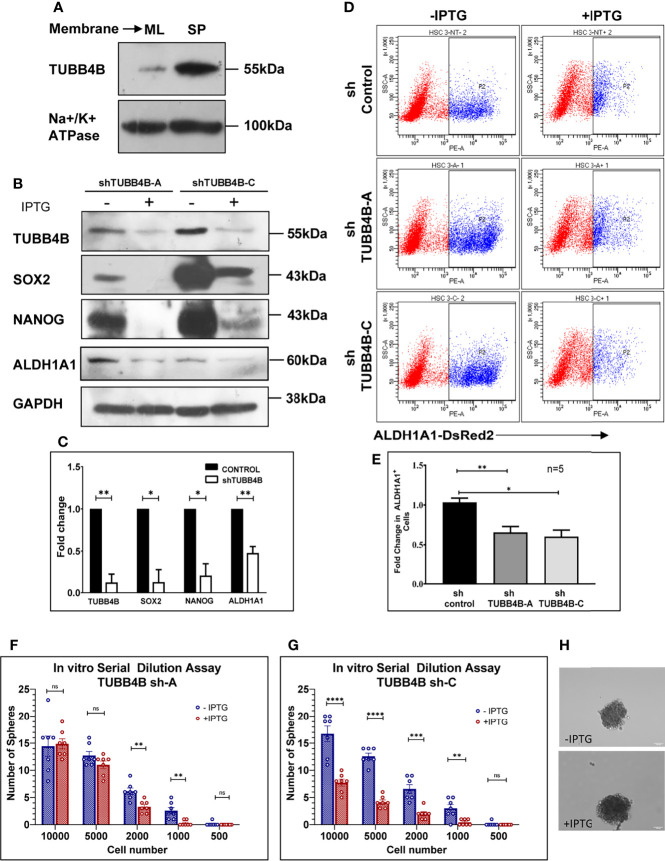
TUBB4B supports CSCs. **(A)** Expression analysis of TUBB4B in the membrane fraction of monolayer (ML) and sphere culture (SP) of HSC-3 cell line by western blotting. **(B)** HSC-3 cells harboring an inducible shRNA (A or C) for TUBB4B were either treated or untreated with 50mM IPTG for six days for shRNA induction, and used for western blot assay of the indicated proteins. **(C)** shows the graphical representation of the normalized protein expressions. **(D)** HSC-3 cells stably expressing ALDH1A1-DsRed2, transduced either of the two TUBB4B shRNA lentiviral particles, or a non-target shRNA were used for the assay. The shRNA expressions were induced by administering 50mM IPTG for 10 days. Induced and uninduced cells were trypsinized, and DsRed2 expression was analyzed by FACS. Subpopulation P2 indicated high ALDH1A1 expressing (ALDH1A1^+^) cancer stem cells. The fold change of P2 subpopulation in the induced cells with respect to uninduced cells for the different shRNA groups were graphically represented **(E)**. Serial dilutions of TUBB4B shRNA induced and uninduced cells were plated in low adhesion plates in replicates and monitored for sphere formation for 10 days. **(F, G)** are the graphical representations of number of spheres formed using TUBB4B shRNA-A and C respectively. Representative images of spheres formed in induced and uninduced groups are also shown **(H)**. Scale bar is 100μm. The error bars indicate SEM of biological replicates. ns represent non-significant data, * represents p < 0.05, ** represents p < 0.01, *** represents p < 0.001, and **** represents p < 0.0001.

Since anchorage-independent 3D sphere culture is known to enrich the stem cell population, to discover the proteins constituting the niche of CSCs, we previously surveyed the differentially expressing proteins in the membrane fractions of sphere culture against monolayer culture by LC-MS/MS, where we detected TUBB4B enrichment in sphere culture membrane fraction (data not shown). To confirm the observation, we isolated the membrane fractions of sphere and monolayer cultures and examined TUBB4B expression. We observed that there was a significant enrichment in the membrane expression of TUBB4B in the sphere culture in contrast to the monolayer (**Figure 1A**). To evaluate the dependence of stemness on TUBB4B, we screened the expressions of stemness markers upon downregulation of TUBB4B using IPTG-inducible lentiviral shRNA. Significant reductions in the expressions of SOX2, NANOG, and ALDH1A1 were observed upon TUBB4B downregulation (**Figures 1B, C**). Further, we checked the expression of ALDH1A1 by immunostaining and confocal microscopy upon TUBB4B downregulation using siRNA. Our results showed a marked reduction in the ALDH1A1^+^ cells when TUBB4B was downregulated. (**Supplementary Figure 1**). Further, we used the IPTG inducible lentiviral TUBB4B shRNA constructs in HSC-3 cells expressing ALDH1A1-DsRed2, a confirmed reporter for CSCs in oral cancer (25). There was a significant reduction in the DsRed2 expressing population upon downregulation of TUBB4B (**Figures 1D, E**). We have earlier shown that DsRed2^+^ cells have higher ALDH1A1 expression, the activity of ALDH, expression of pluripotency markers, soft agar colony formation efficiency, and tumor initiation capacity (25). Here, we have validated the ALDH1A1 DsRed2 construct in HSC-3 cell line using Aldeflour assay. 72% of the ALDH1A1 DsRed2 high expressing population had a high ALDH activity. Of the low expressing population, only 10% had high ALDH activity (**Supplementary Figure 2**). We further observed that though there was no change in the sizes of spheres formed, there was a significant reduction in the number of spheres formed in TUBB4B downregulated group when we performed an *in vitro* serial dilution assay spheroid formation. There was a concurrent reduction in stem cell frequency as calculated using ELDA (Extreme limiting dilution Analysis) (26) (**Figures 1F–H**). The CSC frequency reduced from 1:646 cells to 1:1500 upon downregulation in cells with shRNA-A construct, with a p-value of 0.05, whereas it fell from 1:593 to 1:1326 when downregulation was induced in cells with shRNA-C construct, with a p-value of 0.059. When taken together, these results suggested a CSC regulating role for TUBB4B.

### TUBB4B Downregulation Leads to a Reduction in Tumor Initiation Ability *In Vivo*

**Figure 2:**
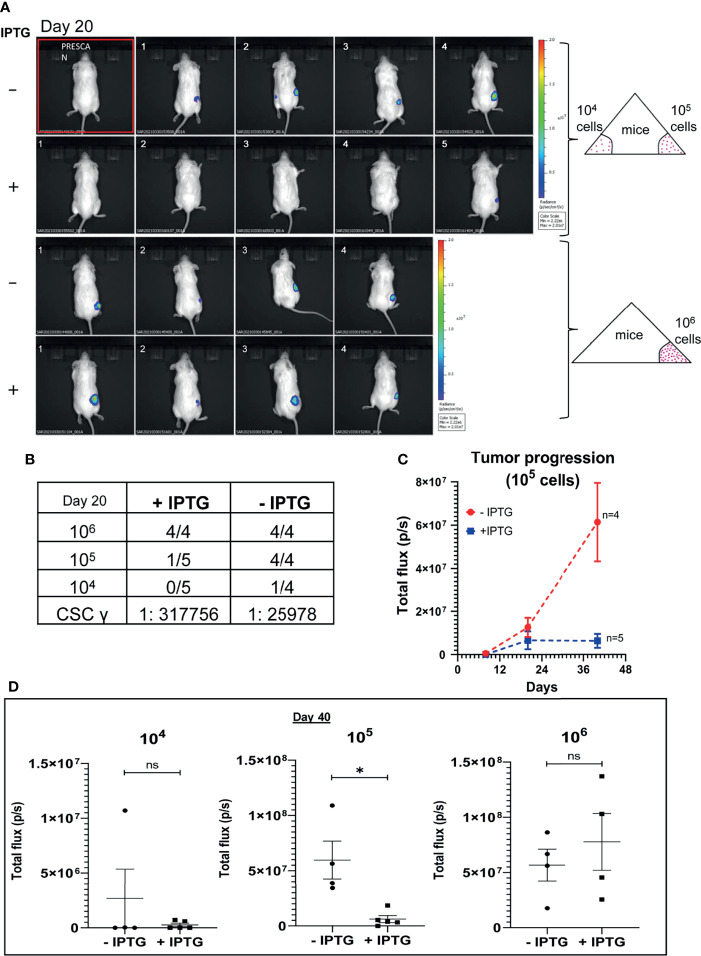
TUBB4B downregulation affects tumor initiation potential in mice xenograft. HSC-3 cells stably expressing Luciferase gene were transduced with lentivirus carrying inducible TUBB4B shRNA. The cells were split into two groups, and either induced with 50mM IPTG for 15 days to downregulate TUBB4B or were left uninduced. Post validation of downregulation by western blotting, serial dilutions of cells, 10^4^, 10^5,^ and 10^6^ were injected into flanks of NOD.CB17-*Prkdc^SCID^*/J mice to generate xenografts. IPTG treatment was continued for another seven days for the induced animals. The tumor growth was assessed by measuring the bioluminescence at days 8, 20, and 40 post the cell injection. **(A)** shows the bioluminescence image and **(B)** summarizes the tumor initiation observed 20 days post cell injections along with the CSC frequency calculated using ELDA. A total flux minimum value of 2x10^6^ p/s was considered as the cutoff for tumor detection. **(C)** is a graphical representation of the tumor progression observed over a period of 40 days in the animals injected with 10^5^ cells. **(D)** is the graphical representation of the luminescence in total flux measured in the different dilution groups at day 40. ns represent non-significant data, and * represents p-value < 0.05.

To further validate the implications of CSC regulatory role of TUBB4B observed in the *in vitro* experiments, an *in vivo* serial dilution xenograft assay was performed with 10^6^,10^5^ and 10^4^ cells of TUBB4B shRNA induced and uninduced groups. Although there was no change in the tumor initiation efficiency on day 20 between the un-induced and induced animals grafted with 10^6^ cells, there was a significant reduction in the tumor initiation potential in the groups with lower cell numbers (10^5^ and 10^4^) (**Figures 2A, B**). Statistical significance of tumor-initiating potential in the two groups was analyzed by ELDA, which computed the stem cell frequency in the uninduced group as 1:25,978, in contrast to 1:3,17,756 in the TUBB4B downregulated group with a p-value of 0.00422. Even though the difference in tumor initiation efficiency was evident on day 20, the effect of the downregulation on tumor progression could not be assessed at this time point. There was a significant delay in the tumor progression in the IPTG-induced 10^5^ cells group as the tumor progressed to day 40 (**Figure 2C**). On day 40, there was a significant reduction in the tumor burden, evidenced by the total flux, in the TUBB4B depleted 10^5^ group, which was not evident in the 10^6^ groups (**Figure 2D**). These results indicate that TUBB4B could be one of the regulators of *in vivo* tumor initiation potential, which is a hallmark of CSCs.

### TUBB4B Supports the CSC Niche in Oral Cancer

**Figure 3:**
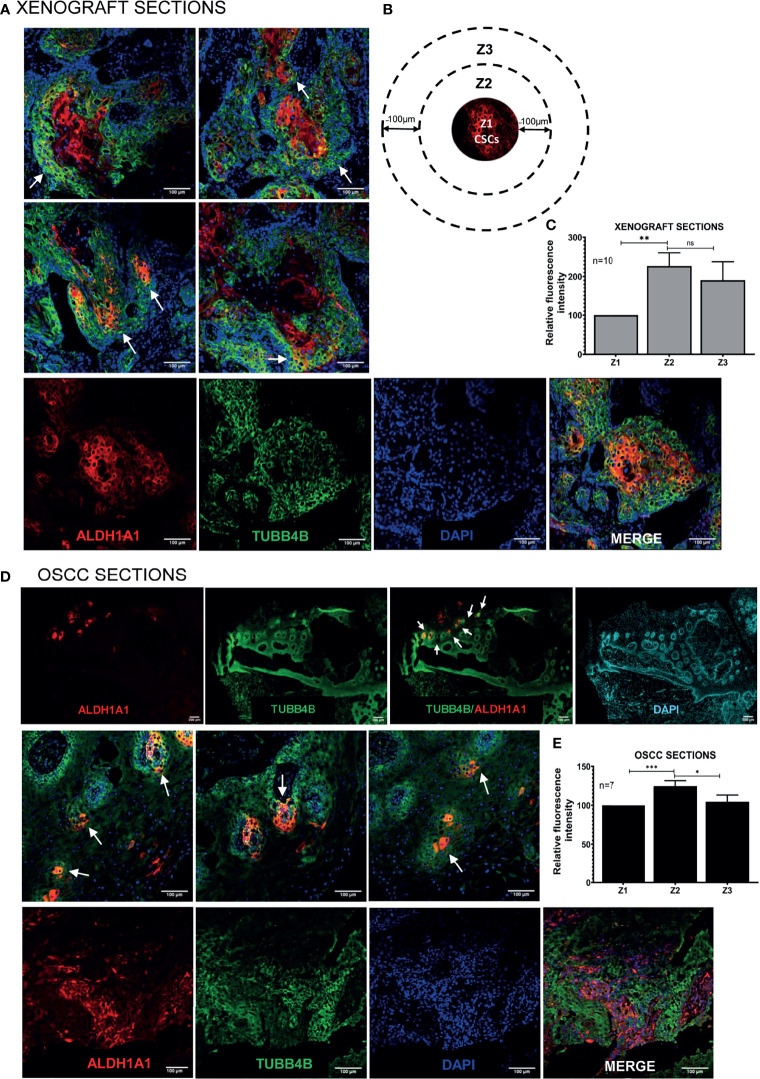
TUBB4B is present in the CSC niche of Oral Cancer. **(A)** HSC3 xenograft sections were immunostained with antibodies against ALDH1A1 and TUBB4B, and confocal images were acquired. The region in and around ALDH1A1^+^ was arbitrarily divided into three zones, with Z1 representing the ALDH1A1^+^ stem cell population, Z2 representing the cells immediately surrounding 100μm from Z1, and the region 100 μm further away from Z2 were considered as zone 3, schematically represented in **(B)**. Ten sections were stained and, three frames were captured from each section. Fluorescence intensities were measured by drawing ROIs in each zones and normalized intensity values were represented graphically **(C)**. The experiment was repeated with seven OSCC sections. **(D)** shows full field stitched images and 20X images. The normalized fluorescence intensity values were plotted **(E)**. Scale bar represents 100μm for all 20X images and 200μm for stitched images. Arrows point towards regions of increased TUBB4B expression in CSC niche. ns represents non-significant data, * represents p < 0.05, ** represents p < 0.01, *** represents p < 0.001.

To investigate the correlation of TUBB4B to CSCs, we did an immunohistochemical analysis of TUBB4B and ALDH1A1 on the xenograft sections, which revealed that TUBB4B expression is high in the areas immediately surrounding the ALDH1A1^+^ cells (**Figure 3A**). For quantification, since the cancer stem cell niches are not defined well in any experimental models, we have taken normal stem cell niches to define our CSC niche. Normal stem cell niches in intestinal crypts (11), hair follicle (27), and hepatic stem cell niche (28) are all supported by approximately 5-10 cells surrounding the stem cells. As the average size of an oral keratinocyte is 20 μm, the total niche size for ten cells, five cells on either side will be 100 μm. So we have taken Zone1 as the high ALDH1A1 expressing CSCs, Zone 2, 100µm outside Zone 1 as the CSC niche, and Zone 3 as the region outside the niche (**Figure 3B**). Our analysis on xenograft sections showed that the highest expression of TUBB4B was in Zone 2, the area immediately surrounding the ALDH1A1^+^ population (**Figure 3C**). Similar experimental analysis with the tissue sections of oral squamous cell carcinoma (OSCC) corroborated with the results obtained from the xenograft sections further confirming the role of TUBB4B in constituting a CSC niche in oral cancer (**Figures 3D, E**).

### Ephrin-B1 Is a Decisive Factor in Predicting the Survival of Patients With Varying Levels of TUBB4 Expression

**Figure 4:**
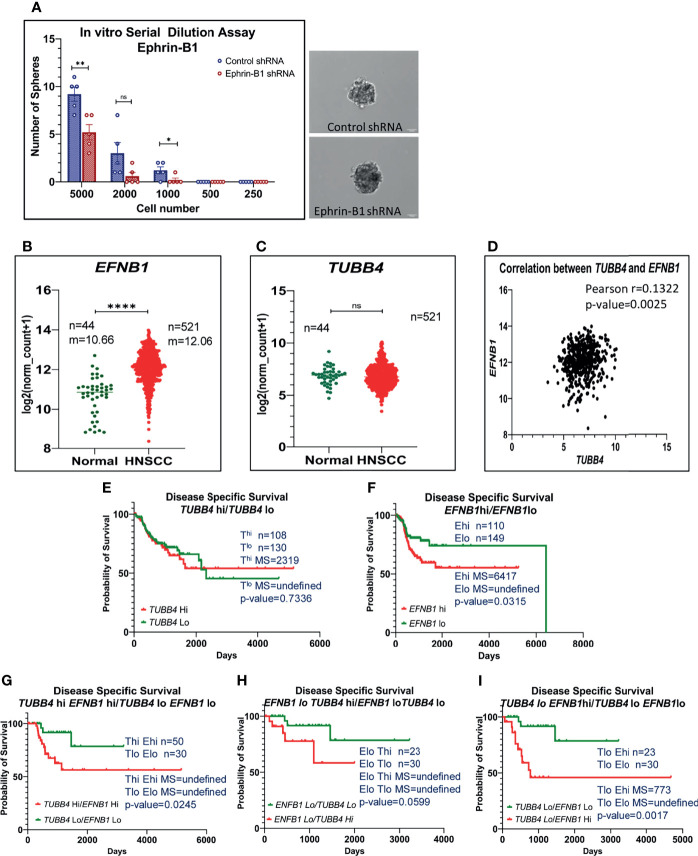
High expression of TUBB4B with Ephrin-B1 predicts poor prognosis in HNSCC. Serial dilutions of control shRNA induced and Ephrin-B1 shRNA harboring HSC3 cells were plated in low adhesion plates in replicates and monitored for sphere formation for 10 days. The number of spheres formed in each dilutions of both groups are represented in the bar graph **(A)**. Representative images of spheres formed in control shRNA and Ephrin-B1 shRNA groups are also shown. Scale bar is 100μm. To analyze mRNA expressions, log2(x+1) transformed RSEM normalized count of *EPHB1* **(B)** and *TUBB4B* **(C)** from 521 HNSCC patients was plotted against 44 normal samples obtained from RNAseq data of TCGA database was performed by using GraphPad prism 9.0. The statistical significance was calculated by unpaired t-test. **(D)** Two-tailed Pearson correlation analysis between *TUBB4B* and *EFNB1* RNA normalized counts of 521 HNSCC patients was performed. Kaplan–Meier analysis was carried out between **(E)** *TUBB4B* ^hi^(>7.5) and *TUBB4B* ^lo^(<6) samples, **(F)** *EFNB1* ^hi^(>12.5) and *EFNB1* ^lo^(<11.5) samples **(G)** *TUBB4B* ^hi^(>7.5)*EFNB1* ^hi^(>12.5) and *TUBB4B* ^lo^(<6)*EFNB1* ^lo^(<11.5) samples **(H)** *EFNB1* ^lo^(<11.5)*TUBB4B* ^hi^(>7.5) and *EFNB1* ^lo^(<11.5)*TUBB4B* ^lo^ **(I)** *TUBB4B* ^lo^(<6)*EFNB1* ^hi^ (>12.5) and *EFNB1* ^lo^(<11.5)*TUBB4B* ^lo^(<6). The statistical significance was calculated by Log-rank (Mantel-Cox) test. MS represents Median Survival. ns represents a non-significant difference between the two data, * represents p < 0.05, ** represents p < 0.01, **** represents p < 0.0001. ^hi^ represents high mRNA expressing group and ^lo^ represents low expressing groups.

Thus far, we have shown clear evidence of the involvement of TUBB4B in the maintenance of CSC and the presence in CSC niche using *in vitro*, *in vivo* tumor models and cancer tissue sections. However the underlying mechanism still remains ill-defined. Since tubulins by themselves are not reported to be involved in signaling events, we hypothesized that it is cooperating with other molecules in the niche to aid CSC maintenance. As Ephrin family of proteins are reported to be a part of the intestinal stem cell niche, and as we observed an up-regulation of Ephrin-B1 in the membrane fractions of cells growing in CSC-enriching culture conditions previously by LC-MS/MS (data not shown), we tested the possible cooperation of TUBB4B and Ephrin-B1 in oral cancer. We confirmed the CSC maintenance function of Ephrin-B1 by *in vitro* serial dilution assay of Ephrin-B1 downregulated cells. We observed a significant reduction in the average number of spheres formed in cells expressing Ephrin-B1 shRNA as compared to cells expressing control shRNA, though there was no change in size of spheres formed (**Figure 4A**). The CSC frequency, calculated using ELDA online tool was 1:646 for cells expressing control shRNA, whereas that of Ephrin-B1 shRNA expressing cells was found to be 1:1500, with a p-value of 0.0297. To test out cooperation between TUBB4B and Ephrin-B1 in oral cancer, we initially checked whether the expressions of these proteins are involved in the progression of oral cancer using data retrieved from the TCGA repository (29). Our analysis of the RNA-seq data revealed an up-regulation of *EFNB1* in the OSCC samples compared to normal samples, while the expression of *TUBB4* was unaltered (**Figures 4B, C**). Moreover, the correlation analysis ruled out transcriptional interdependence or any common upstream regulators (**Figure 4D**). Though the high expression of *TUBB4* alone was not a predictor for Disease specific survival, the simultaneous increased expressions of both *TUBB4* and *EFNB1* predicted a poor survival compared to the concurrent low expressions of these two proteins (**Figures 4E–G**). Also, a high expression of at least one of the proteins decreases the survival when compared to patients with low expressions of both the proteins (**Figures 4H, I**). Patients with high *EFNB1* expression had poor survival compared to *TUBB4*, as is evident from the plot patterns in **Figures 4E, F**. Further, *TUBB4* ^Hi^+*EFNB1* ^Lo^ subset had a survival advantage over the *TUBB4* ^Lo^+*EFNB1* ^Hi^ subset, as evidenced by the very low median survival of 773 days in the latter group (**Figures 4H, I**). When taken together, analysis of the clinical data points out that TUBB4 and Ephrin-B1 might have functional cooperation, primarily depending on Ephrin-B1, which affects the tumor properties deciding survival of the patients.

### Ephrin-B1 Interacts With TUBB4B on the Cell Membrane of the Cells in the CSC Niche

**Figure 5:**
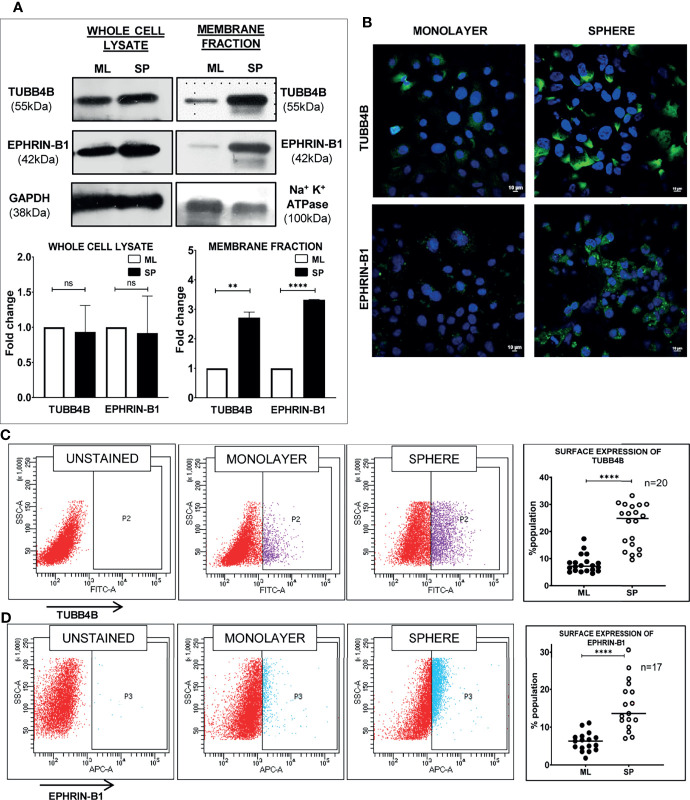
Sphere culture enriches the surface expression of TUBB4B and Ephrin-B1. **(A)** Whole-cell lysates and membrane fractions of sphere and monolayer cultures of HSC-3 cells were immunoblotted. For whole-cell lysates, GAPDH was used as loading control, whereas Na^+^/K^+^-ATPase was used as loading control for membrane fractions. The fold changes were calculated and represented graphically. **(B)** Shows the membrane expressions of TUBB4B or Ephrin-B1 in unpermeabilized monolayer (ML) and sphere cultures (SP). PFA fixed HSC3 sphere or monolayer cultures were probed for **(C)**TUBB4B or **(D)** Ephrin-B1 using fluorophore tagged primary antibodies and the surface expressions were analyzed by flow cytometry. The graphs represent the percentages of cells with high surface expression of individual proteins. ns represents non-significant data, ** represents p < 0.01, **** represents p < 0.0001.

**Figure 6:**
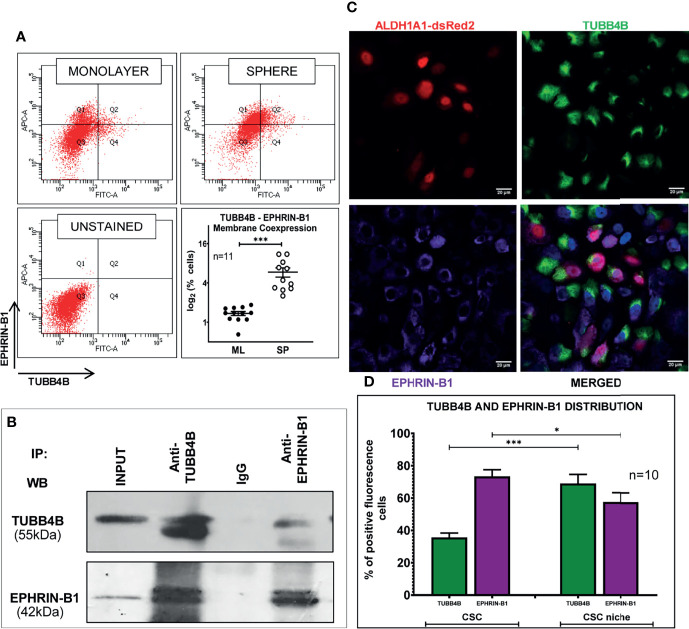
TUBB4B and Ephrin-B1 physically interact in the CSC niche. **(A)** PFA fixed monolayer (ML) and sphere (SP) cultures of HSC-3 cells were dual stained with fluorophore tagged TUBB4B and Ephrin-B1 antibodies and the percentage dual positive cells were plotted. **(B)** Lysates of HSC-3 cells were immunoprecipitated with TUBB4B antibody and a reverse IP was performed using Ephrin-B1 antibody. The elutes were separated by SDS PAGE, transferred and probed for the indicated proteins. Isotype control mouse IgG antibody was used as the negative control. **(C)** Unpermeabilized HSC-3 cells with ALDH1A1-DsRed2 reporter were probed with TUBB4B and Ephrin-B1 antibodies, and confocal images were acquired. Cells with high fluorescence intensities were manually counted for each channel in ALDH1A1^+^ cell clusters (the CSC population), and in the cells immediately surrounding the ALDH1A1^+^ cell, within a distance of 100 μm (considered to be the CSC niche cells). Of the total number of CSCs or the niche cells, the percentages of cells with high expression of each protein were plotted **(D)**. Cells were counted from 10 frames. * represents p-value < 0.05 and *** represents p < 0.001. Scale bar represents 20 μm.

**Figure 7:**
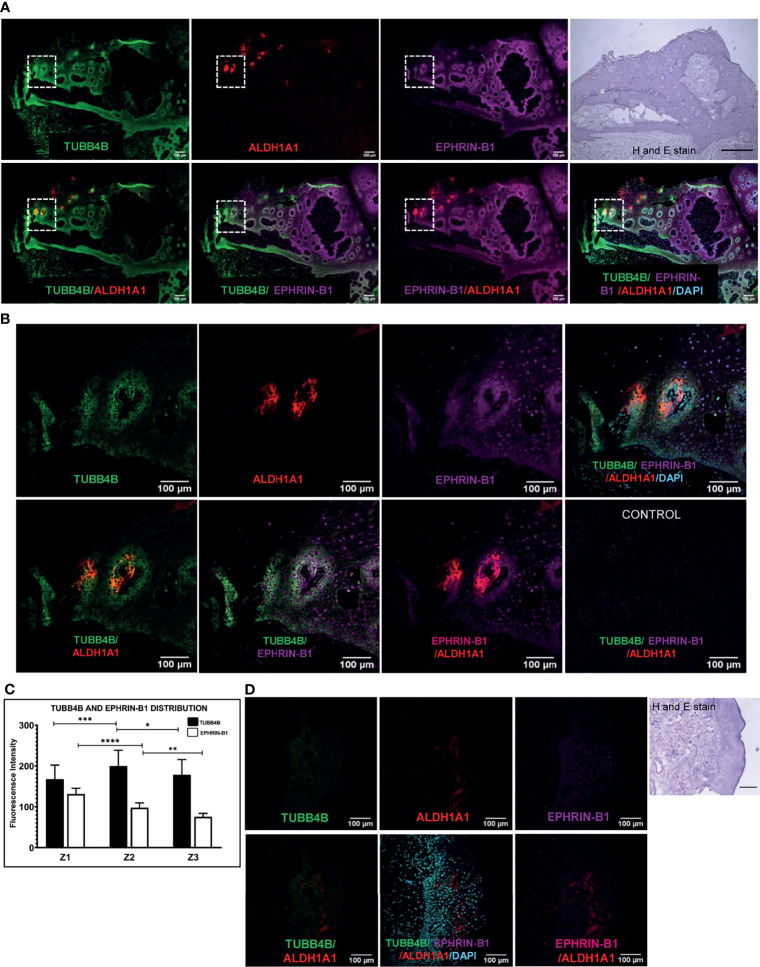
TUBB4B and Ephrin B-1 co-express in CSC niche of Oral cancer. Sections of OSCC patient tumors were triple stained with ALDH1A1/TUBB4B/Ephrin-B1. **(A)** shows stitched full field images of the tissue sections at 10X magnification. The scale bar represents 200µm. Hematoxylin and Eosin staining of the same tissue is also shown, for which scale bar represents 500 µm. The areas in the inset are shown at a magnification of 20X in **(B)**. The scale bar represents 100 µm. For quantitation, the region in and around ALDH1A1^+^ was arbitrarily divided into three zones, with Z1 representing the ALDH1A1^+^ stem cell population, Z2 representing the cells immediately surrounding 100μm from Z1, and the region 100 μm further away from Z2 were considered as zone 3. Fluorescence intensities were measured by drawing ROIs in the zones and represented graphically **(C)**. **(D)** shows 20X images of ALDH1A1/TUBB4B/Ephrin-B1 triple staining of normal tissue. * represents p < 0.05, ** represents p < 0.01, *** represents p < 0.001, **** represents p < 0.0001.

**Figure 8:**
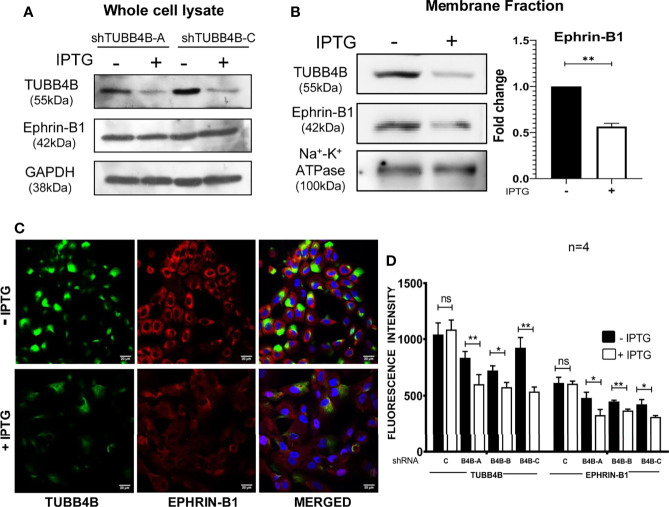
TUBB4B downregulation reduces Ephrin-B1 on the surface. HSC-3 cells stably expressing inducible TUBB4B shRNAs were induced with 50mM IPTG for ten days. Whole-cell lysates **(A)** and membrane fractions **(B)** of the TUBB4B shRNA induced and uninduced cells were probed for the indicated proteins by immunoblotting. **(C)** non-permeabilized TUBB4B shRNA induced and uninduced cells were immune-stained for the indicated proteins. The fluorescence intensities of TUBB4B and Ephrin-B1 were calculated by drawing ROIs around cells. Average intensities were graphically plotted for induced and uninduced cells of the three lentiviral TUBB4B shRNAs (B4B-A, B4B-B and B4B-C), and non-target shRNA(c). ns represent non-significant data, **(D)** * represents p < 0.05, ** represents p < 0.01. Scale bar represents 20 μm.

When the clinical data suggested a functional interaction between TUBB4B and Ephrin-B1 regulating survival, we sought to investigate how they cooperate to regulate CSCs in oral cancer. Since the expression of Ephrin-B1 on the cell surface is vital for its signaling function, we looked at the surface expression in HSC-3 cell line. Our western blot analysis showed that the expression of both TUBB4B and Ephrin-B1 are up-regulated on the cell membrane of CSC-enriched sphere culture cells, even when the total expression was not significantly different from that of monolayer cells. We obtained similar results in another oral cancer cell line, SAS (**Figures 5A** and **Supplementary Figure 3**). Our immunofluorescence analysis and FACS analysis confirmed the enrichment of cells expressing TUBB4B or Ephrin-B1 on the cell surface in sphere cultures of oral cancer cell lines (**Figures 5B–D** and **Supplementary Figures 4A, B**). Further, we show that an increased number of cells co-express these proteins on their surface in sphere culture (**Figure 6A** and **Supplementary Figure 4C**). Moreover, our protein pull-down with anti-TUBB4B from HSC-3 cell lysate co-precipitated Ephrin-B1. The coexistence of TUBB4B and Ephrin-B1 in a functional complex was confirmed by reverse immunoprecipitation (IP) using anti-Ephrin-B1 antibody (**Figure 6B**). IP and reverse IP results were reproduced in SAS cell line (**Supplementary Figure 4D**). We also observed surface co-localization of TUBB4B and Ephrin-B1 in different oral cancer cell lines (**Supplementary Figure 4E**). The relevance of this complex formation in the CSC niche context was evaluated in HSC-3 cells by immunostaining. The expression of TUBB4B was significantly higher in the CSC niche than in the CSC population, whereas there was a high expression of Ephrin-B1 in both CSCs and CSC niche (**Figures 6C, D**). The expressions of these proteins in relation to ALDH1A1^+^ population in OSCC sections checked by triple staining of TUBB4B/ALDH1A1/Ephrin-B1 suggested that a high expression of Ephrin-B1 is observed in the CSC population and in the CSC niche, and is also seen to co-express with TUBB4B in the CSC niche cells (**Figures 7A–C**). We have also checked the expressions of the three proteins in tumor adjacent normal tissue where we observed decreased expressions (**Figure 7D**). To check whether the expression of Ephrin-B1 is dependent on the expression of TUBB4B, we used the TUBB4B knockdown cells. While the total expression of Ephrin-B1 was not affected by the TUBB4B depletion (**Figure 8A**), its surface localization was significantly reduced as seen from western blot analysis of Ephrin B1 expression in the membrane fraction, as well as immunofluorescence assisted visualization of surface expression of Ephrin-B1 (**Figures 8B, C** and **Supplementary Figure 5**). Using a non-targeted control and three different shRNAs of TUBB4B and siRNA, we have confirmed that the surface expression of Ephrin-B1 relies on the expression of TUBB4B (**Figure 8D** and **Supplementary Figure 5**).

## Discussion

The multi tubulin hypothesis states that individual tubulin genes might encode functionally divergent polypeptides that may confer unique properties to the final microtubule polymer (30). Evidence has been accumulating for and against this hypothesis for the last decade (31, 32). The significance of microtubules in mitosis and cell division makes them a promising target for cancer chemotherapy (33). Consistent with the overexpression of specific isotypes of tubulins in cancers, the success of several microtubule-targeting agents have been compromised (34–37). Overexpression of both βII tubulin and βIII tubulin are reported in cancer (24, 38). βII tubulin is shown to regulate Voltage-Dependent Anion Channels (VDACs) in the mitochondrial outer membrane, which act as a critical regulator of the Warburg effect observed in cancer cells (39, 40). At the same time, overexpression of βIII-tubulin (TUBB3) is associated with the development of resistance to microtubule-targeting agents, resistance to apoptosis, and development of metastasis (41). Also, nuclear localization of βII tubulin is associated with poor outcomes in cancers (38). Even though the expression of TUBB4B was evaluated in some studies, its role in the regulation of oral cancer CSCs is not well explored in oral cancer.

As the high membrane expression of TUBB4B on the sphere cells, a population enriched with CSCs suggested its possible role in maintaining the CSCs, we tested whether its downregulation will diminish the overall CSC count. Our results showed that the CSC population and the stemness markers reduced upon depletion of TUBB4B. Consistent with that, the tumor initiation potential *in vitro* and *in vivo* of TUBB4B knocked-down cells was significantly low. Since tumor growth is regulated by different factors other than tumor initiation potential, the difference in tumor mass between the two groups in the highest cell number was not evident. In the *in vivo* condition, both in xenografts and OSCC sections, the expression of TUBB4B was in and around the CSC population, suggesting its role in constituting a CSC niche. Studies conducted to identify the regulation of CSC niche in the maintenance of self-renewal and differentiation in the normal stem cell compartments have shown that generation of expression gradients of different signaling molecules, like WNT, BMP, Eph receptors, and Ephrin-B1 play a role in the intestinal crypt cell homeostasis (7–11). Eph receptors and Ephrins are also reported for the maintenance of other adult stem cell niches like that of mammary gland, neural, skin and prostate (42–44). Eph/Ephrin signaling is also shown to be involved in cancer stem cell niche maintenance in leukemia and Glioblastoma (45–47).

Since the reported functions of microtubules do not support its direct involvement in imparting stemness by transcriptional activation or inducing a signaling pathway to induce the property, we looked for proteins that TUBB4B might be cooperating to constitute a niche. Ephrin-B1, a protein reported to regulate intestinal stem cell niche, was observed to be up-regulated in self-renewing conditions, like sphere culture. We investigated the possible functional interaction of these proteins in oral cancer patient samples using data retrieved from TCGA. The expression analysis and correlation analysis ruled out a synchronized regulation of the two proteins. But the Kaplan-Meier survival analysis suggested functional cooperation of these two proteins in the aggressiveness of the disease as the high expression of both these proteins or increased expression of even one of these proteins can significantly lead to poor prognosis. It is worthwhile to mention here that, like TUBB4B, its close relatives TUBB2 and TUBB3 have been shown to predict poor survival, the former regulating the cancer energetics through VDAC and the latter by imparting resistance to microtubule targeting agents (24, 38–41). Our results show that the mechanism by which TUBB4B influences survival is through another means, possibly by regulating the proper localization of Ephrin-B1 in the CSC niche.

We have evaluated the co-expression of these two proteins on the cell surface by FACS and immunofluorescence *in vitro*, and its significance *in vivo* was assessed using OSCC sections. Our results showed that Ephrin-B1 and TUBB4B co-localize maximally in the CSC niche, suggesting that TUBB4B is probably involved in the surface localization of Ephrin-B1 to create a gradient in a CSC niche, which is essential for the support of the CSCs. Their physical interaction was confirmed by immunoprecipitation with anti-TUBB4B and reverse-immunoprecipitation with Ephrin-B1. Further, we showed that the surface expression of Ephrin-B1 is dependent on TUBB4B using shRNA-mediated knockdown. Hence, TUBB4B directly regulates the membrane expression of Ephrin-B1, which in turn regulates the signaling in CSC niche. Alteration of TUBB4B expression leads to a decrease in surface expression of Ephrin B1, which causes an imbalance in CSC-niche signaling, resulting in a fall of CSC population, which is reflected in the reduction in ALDH1A1^+^ cells. There has been a previous report that TUBB4 is necessary for the proper membrane localization of newly expressed mesenchymal marker N-cadherin (48). Their results also suggest that downregulation of TUBB4 makes the microtubules shorter, and they often do not connect to the cell membrane in cells undergoing endothelial mesenchymal transition. A similar trafficking function of TUBB4B is also shown for the expression of another cell surface protein, GLUT1, which is critical for regulating stemness (14).

The process of self-renewal and differentiation in the CSC niche is governed by specific signaling pathways which are in turn controlled by the spatiotemporal regulation of the expression of specific ligands and receptors. This orchestrated expression might be achieved by specific membrane transporters like TUBB4B. Ephrin B1 is an important molecule regulating the self-renewal/differentiation of stem cells, but it could be only one of the many molecules transported by TUBB4B in the CSC microenvironment.

In conclusion, our findings indicate that β-tubulin isotype TUBB4B is essential for maintaining the stemness of cancer stem cells. We also conclude that this function can be attributed to tubulin regulating the membrane localization of proteins critical for stem cell maintenance signaling. We speculate that the effect of TUBB4B on CSC niche maintenance by altering Ephrin-B1 localization could be by a microtubule-dependent trafficking process, the dynamics of which are fine-tuned by the unique C-terminal sequence of TUBB4B; or could also be a result of binding of free monomeric TUBB4B to Ephrin, the possibility of which could be explored in the future.

A recent study proposed TUBB4B as a potential marker in Ovarian Cancer, wherein they report an increased level of TUBB4B in ovarian cancer patient plasma samples in comparison to healthy and benign non-cancerous samples. They also observed a direct correlation between the levels of TUBB4B and the stage of cancer progression (21). A Glioblastoma study has proposed small molecular inhibitors targeting TUBB4 as adjuvant therapy in Glioblastoma treatment, as they observed a fall in GLUT1 expression, a protein essential for Glioblastoma stem cell survival. As our study demonstrates relevance of TUBB4B in Oral cancer stem cell niche, we believe targeting TUBB4B using specific small molecule inhibitors could be a potential aid in clearing the CSC population by creating an imbalance in the CSC niche.

## Data Availability Statement

The raw data supporting the conclusions of this article will be made available by the authors, without undue reservation.

## Ethics Statement

The studies involving human participants were reviewed and approved by Institute Human Ethical Committee, Rajiv Gandhi Centre for Biotechnology (IHEC/1/2011/04) and Human Ethical Committee, Regional Cancer Centre (HEC/24/2011). The patients/participants provided their written informed consent to participate in this study. The animal study was reviewed and approved by Institute Animal Ethics Committee, Rajiv Gandhi Centre for Biotechnology, Thiruvananthapuram.

## Author Contributions

DD and TM have designed the experiments. DD has performed the experiments and written the draft manuscript. AJ has helped in conducting the FACS and animal experiments. AM has developed ALDH1A1-DsRed2 construct. PB, NG, and PS have provided with the oral cancer tissue sample. TM and SS have looked after the experiments and made the final version of the manuscript.

## Funding

The work was supported by the institutional core funding from the Department of Biotechnology to SS and TM, and extramural grant to TM from DST (SR/S0/HS/0133/2010) and DBT (BT/PR14379/Med/30/536/2010). DD is thankful to the University Grants Commission (UGC) (22/06/2014(i)EU-V) and Indian Council of Medical Research (ICMR) (3/2/2/39/2020-NCD-III), Government of India, for fellowship supports.

## Conflict of Interest

The authors declare that the research was conducted in the absence of any commercial or financial relationships that could be construed as a potential conflict of interest.

## Publisher’s Note

All claims expressed in this article are solely those of the authors and do not necessarily represent those of their affiliated organizations, or those of the publisher, the editors and the reviewers. Any product that may be evaluated in this article, or claim that may be made by its manufacturer, is not guaranteed or endorsed by the publisher.
