# Supplementary material for: β-Tubulin Isotype, TUBB4B, Regulates The Maintenance of Cancer Stem Cells
Source: Front Oncol. 2021 Dec 23;11:788024. doi: 10.3389/fonc.2021.788024 (PMC8733585; doi:10.3389/fonc.2021.788024)
Supplement: Supplementary file 1 [file DataSheet_1.pdf]

SUPPLEMENTARY FIGURES

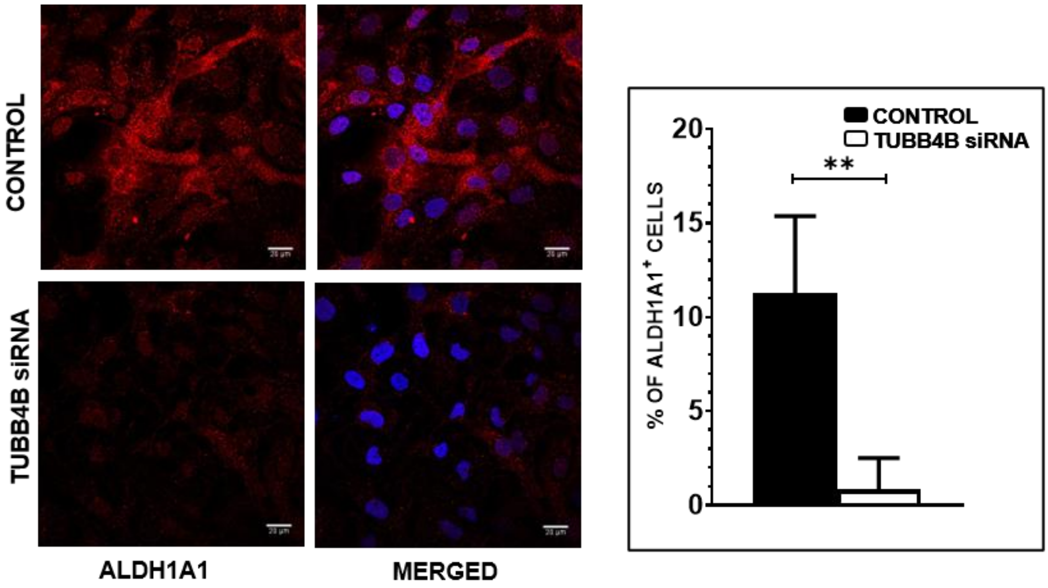

Supplementary Figure 1. Decreased ALDH1A1+ cells upon siRNA mediated TUBB4B downregulation. (A) HSC-3 cells were transfected with control siRNA and 80nM TUBB4B siRNA. Fixed and permeabilized cells were stained with antibody for ALDH1A1. (B)Graphical representation of the percentage of ALDH1A1 high cells. n=118 for control cells and n=149 for TUBB4B downregulated cells. ** represents $p < 0.01$. Scale bar represents 20 µm.

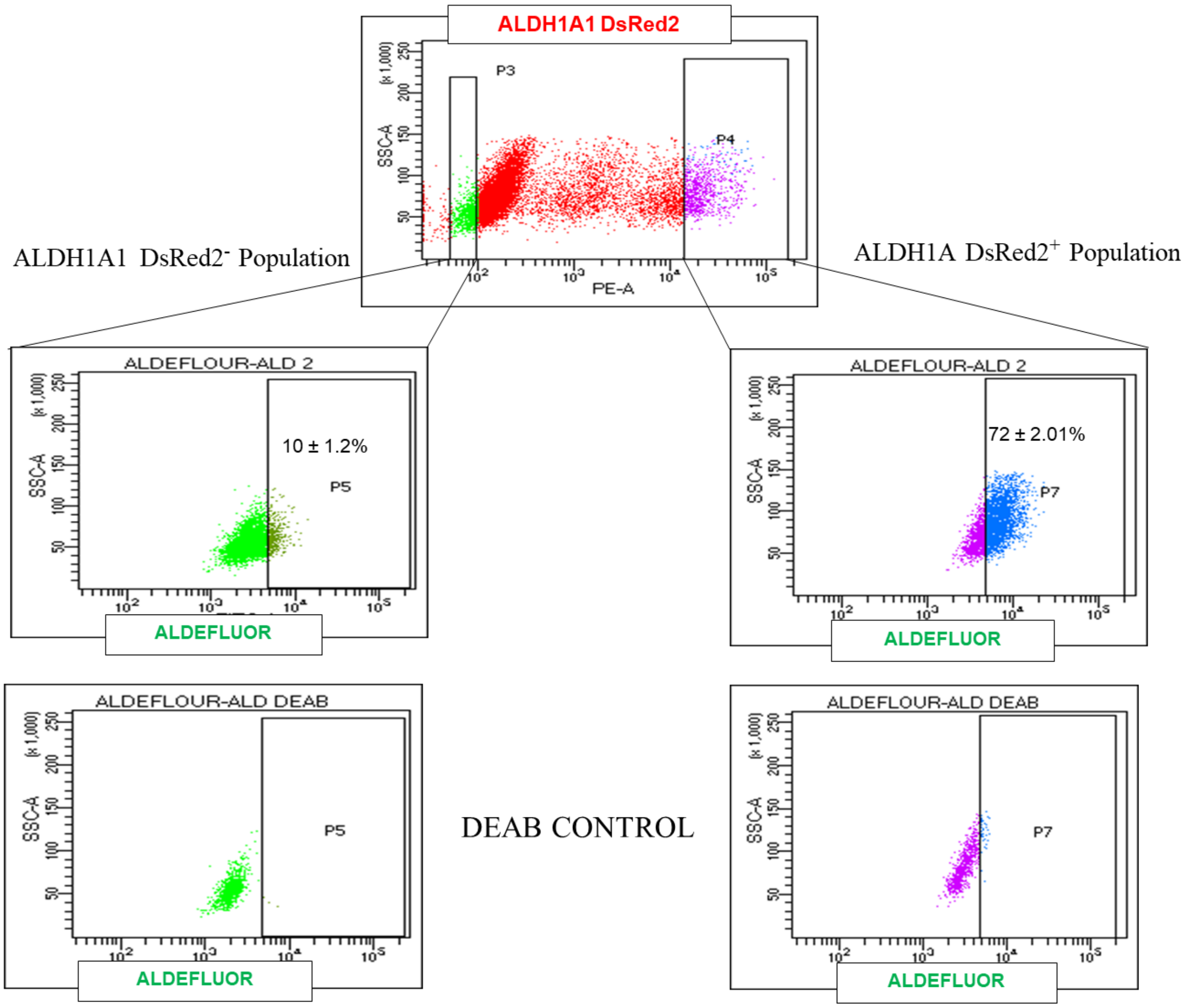

Supplementary Figure 2 Aldefluor Assay to validate the ALDH1A1 DsRed2 construct. Four tubes with $2x10^5$ALDH1A1 DsRed2 cells were resuspended in Aldefluor assay buffer and mixed with equal volumes of Aldeflour reagent. DEAB, inhibitor of ALDH activity was added to one of the tubes. The tube contents were mixed well and incubated at 37ºC for 40 minutes while mixing every 10 minutes. Excess reagent was washed off after the incubation and the cells were analysed by flow cytometry. ALDH1A1-DsRed2 high (ALDH1A1-$DsRed2^+$) and ALDH1A1-DsRed2 low (ALDH1A1-$DsRed2^-$) cells were gated and checked for Aldefluor activity.

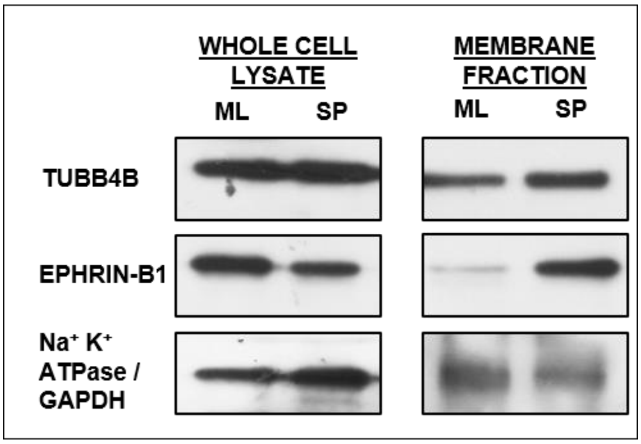

Supplementary Figure 3. Increased membrane expression of TUBB4B and Ephrin-B1 in membrane culture of SAS cell line. Whole cell lysates and membrane fractions of sphere and monolayer cultures were extracted for western blotting and were probed with TUBB4B and Ephrin-B1 antibodies. GAPDH was used as loading control for whole cell lysate samples and Na^+/K^+-ATPase was used as loading control for membrane fractions.

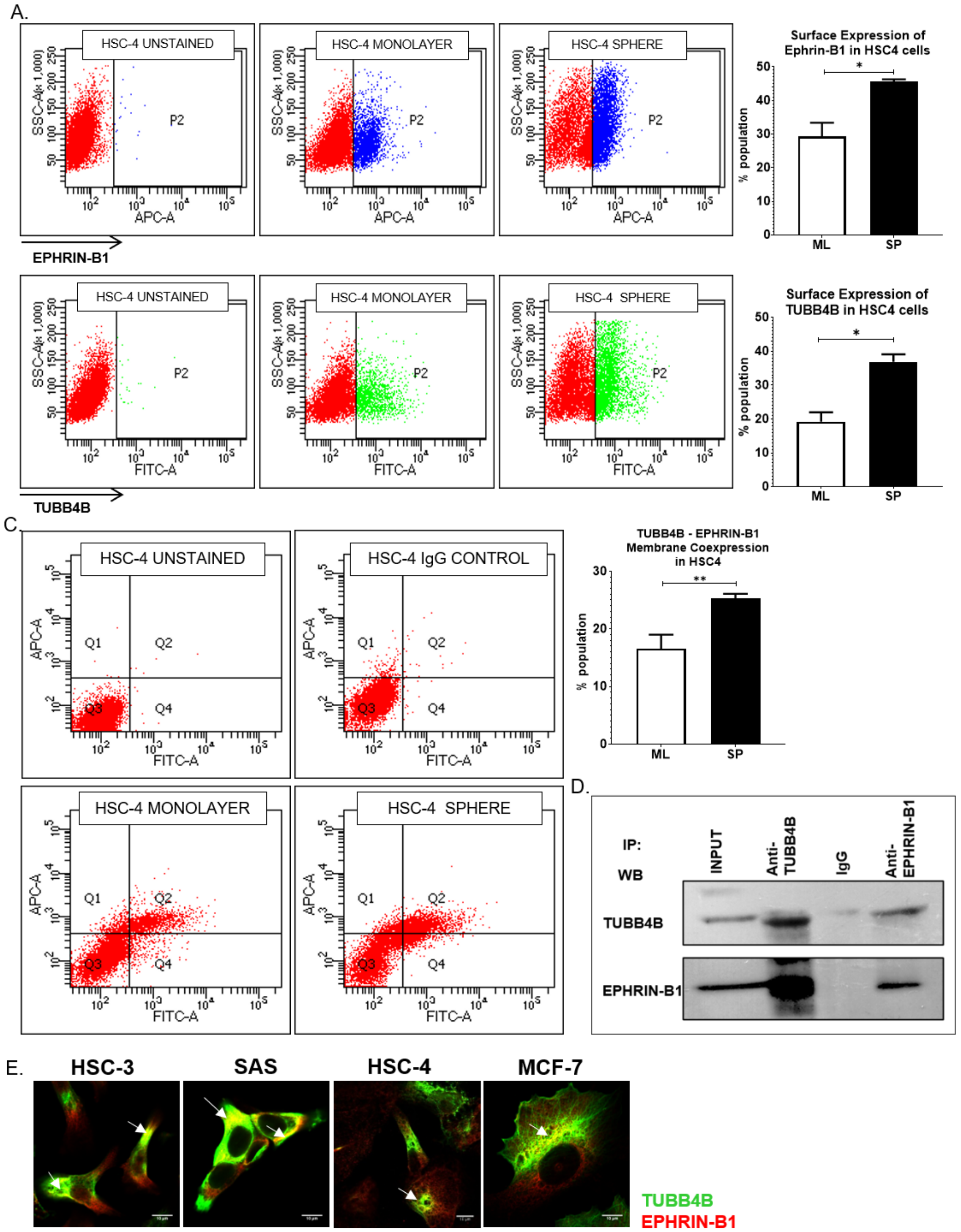

Supplementary Figure 4. PFA fixed HSC3 sphere (SP) or monolayer (ML) cultures were probed for (A)TUBB4B or (B) Ephrin-B1 using tagged primary antibodies and analysed by flow cytometry. (C) PFA fixed monolayer and sphere cultures of HSC-3 cells were dual stained with tagged TUBB4B and Ephrin-B1 antibodies and the percentage dual positive cells were plotted. Immunoprecipitation assay in SAS cell line. TUBB4B was pulled down using antibody and probed for the indicated molecules. Reverse IP was performed by pulling down Ephrin-B1 with antibody and probing for the indicated molecules. Isotype control IgG was used as negative control. Membrane co-staining of TUBB4B and Ephrin-B1 in monolayer cultures of cell lines. Arrows show areas of colocalization. Scale bar represents 10µm.

SAMPLE	AGE	SEX	STAGE	TNM
TM11	38	M	3	T3N1M0
TMAG11	53	M	2	T2N0M0
TM13	75	M	4	T4N0M0
TMAG12	62	M	1	T1N0M0
TM21	50	M	2	T2N0M0
TM20	57	M	2	T2N0M0
TM28	52	M	4	T4N1M0
TM25	50	M	1	T1N0M0
TM30	68	F	4	T4N1M0
TMAG17	51	F	2	T2N0M0
TM29	68	F	1	T1N0M0
TMAG2	45	M	1	T1N0M0

Supplementary Table 1 OSCC Sample details. All the patients were of South Indian origin.

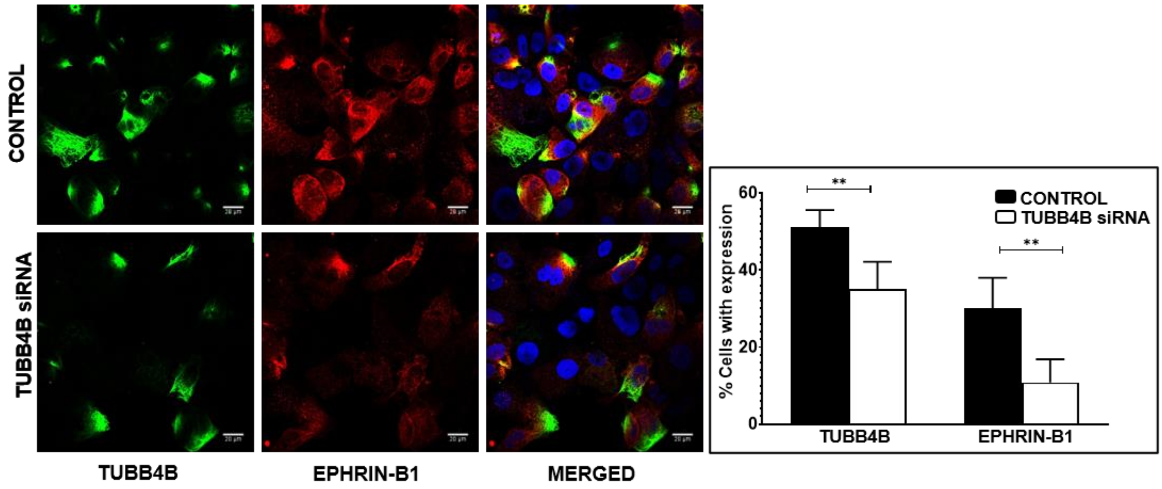

Supplementary Figure 5. Decreased Ephrin-B1 surface expression upon siRNA mediated TUBB4B downregulation. (A) HSC-3 cells transfected with control siRNA and TUBB4B siRNA were fixed and stained for TUBB4B (green) and Ephrin-B1(red). Intensities of TUBB4B and Ephrin-B1 in single cells were measured by drawing ROIs. Average intensities were plotted graphically (B). N=240 for control cells, N=181 for downregulated cells. ** represents $p < 0.01$. Scale bar represents 20 µm.
